# Determination of Biotin Interference in Pediatric Obesity Related ELISA Research Kits Biotin Interference in Manual ELISA Kits

**DOI:** 10.1002/jcla.70079

**Published:** 2025-07-14

**Authors:** Ezgi Kürkçü Kahraman, Orkide Donma, Mustafa Metin Donma, Ahsen Yılmaz, Savaş Güzel

**Affiliations:** ^1^ Department of Nutrition and Dietetics, Faculty of Health Sciences Istanbul Beykent University Istanbul Turkey; ^2^ Department of Biochemistry, Cerrahpaşa Faculty of Medicine İstanbul University‐Cerrahpaşa Istanbul Turkey; ^3^ Department of Child Health and Diseases, Namık Kemal University Faculty of Medicine Tekirdağ Namık Kemal University Tekirdağ Turkey; ^4^ Department of Biochemistry, Namık Kemal University Faculty of Medicine Tekirdağ Namık Kemal University Tekirdağ Turkey

**Keywords:** biotin interference, ELISA, pediatric obesity

## Abstract

**Background:**

Although high‐dose biotin interference in automated immunoassays is now considered, there are very few studies showing biotin interference in manually operated research kits, especially with enzyme‐linked immunosorbent assay (ELISA). The aims of our study were to determine the effects of biotin interference on various parameters, including leptin, leptin receptor (LEPR), ghrelin, acylated ghrelin, deacylated ghrelin, ghrelin receptor (GHSR), kisspeptin (KISS1), kisspeptin receptor (KISS1R), preptin, peroxisome proliferator activated receptor gamma (PPARγ), nod‐like receptor pyrin domain‐containing 3 (NLRP3) and interleukin‐18 (IL‐18), which contribute to energy homeostasis in healthy and obese children.

**Methods:**

Serum pools were prepared from healthy and obese individuals, and biotin concentrations in samples containing different amounts of biotin were measured via sandwich and competitive ELISA methods. In addition, possible biotin interactions were investigated by determining the concentrations of all the study parameters in serum pools containing different amounts of biotin.

**Results:**

We found that the biotin‐competitive, ghrelin‐competitive, KISS1‐competitive, GHSR, leptin and LEPR ELISA kits were less affected by biotin interference and the results of these assay kits were more reliable. Unexpectedly, high levels were also measured in the biotin sandwich ELISA kit, indicating that biotin interference can also occur in manually operated assay kits.

**Conclusions:**

Biotin exhibited an interference effect even in well‐functioning, qualified kits, and this negative effect was less common in competitive kits. Biotin interference was closely associated with the quality of the research kit, the parameters studied, and the presence of high biotin concentrations in the blood.

## Graphical abstract

In this study, we aimed for the first time to demonstrate biotin interference in ELISA research kits used in the analysis of pediatric obesity parameters other than routine testing. Our study revealed that biotin interaction may occur in manual ELISA kits and may cause erroneous test results. It will raise awareness among health professionals, researchers, and medical companies on this issue. By encouraging manufacturers developing medical products to take measures to reduce the effect of biotin interaction, it will prevent erroneous results in scientific studies and contribute to more precise measurements.

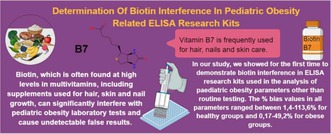

**Abbreviations**
AACC: American Association for Clinical Chemistry
ELISA: Enzyme‐Linked Immuno Sorbent Assay
FDA: The United States Food and Drug Administration
GHSR: ghrelin receptor
IL‐18: interleukin‐18
KISS1: kisspeptin
KISS1R: kisspeptin receptor
LEPR: leptin receptor
max: maximum
min: minimum
NLRP3: nod‐like receptor pyrin domain‐containing 3
PPARγ: peroxisome proliferator activated receptor gamma
WHO: World Health Organization

## 1 Introduction

Biotin, a water‐soluble vitamin, is used as a coenzyme in reactions catalyzed by carboxylase enzymes in gluconeogenesis, fatty acid biosynthesis, single C‐numbered fatty acid degradation, and catabolism of branched‐chain amino acids. Since biotin is also synthesized by intestinal bacteria and taken up through food and diet, its deficiency is rare. The daily dose required for adults is 30 μg. The daily dosage required for biotin in children varies according to age and gender. Children aged 1–3 should receive 8 μg of biotin per day, children aged 4–8 should receive 12 μg per day, children aged 9–13 should receive 20 μg per day, and children aged 14–18 should receive 25 μg of biotin per day [1, 2].

Although biotin deficiency is not common in healthy individuals, it may occur in some hereditary diseases. Metabolic disorders such as biotinidase deficiency, holocarboxylase synthetase deficiency, or biotin‐thiamine‐sensitive basal ganglia disease, long‐term parenteral nutrition, anticonvulsants, or protein pump inhibitors, Crohn's disease, alcoholism, and excessive consumption of raw eggs (avidin binds biotin) may cause biotin deficiency. Biotin treatment can be administered at doses of 5–20 mg/day for biotinidase deficiency and holocarboxylase synthetase deficiency, and 5–10 mg/kg/day for biotin‐thiamine‐sensitive basal ganglia disease. For examples with multiple sclerosis, 300 mg of biotin therapy is prescribed, which is 10,000 times the recommended daily intake [3, 4]. Biotin doses of 5,10,20 mg and 300 mg can cause blood biotin levels of approximately 200–1200 ng/mL [5, 6, 7].

The relationship between biotin and microbiome diversity in severe obesity is very important. In people with severe obesity, circulating biotin levels are below normal, and the expression of biotin‐related genes in adipose tissue varies [8]. Belda et al. showed that biotin supplementation of mice fed a high‐fat diet increased microbiome diversity and improved the potential for bacterial production of biotin and B vitamins while limiting weight gain and glycemic impairment [9]. Furthermore, another study in obese mice showed that biotin supplementation induced beneficial metabolic effects through modulation of lipid metabolism, inflammation, and fibrosis in adipose tissue and liver [10].

The increase in the prevalence of obesity has become an important health issue throughout the lives of children and adolescents as well as adults [11]. Childhood obesity is the cause of adult obesity and is an important problem especially because of its relationship with morbid obesity, metabolic syndrome, diabetes mellitus, cancer, and cardiovascular diseases [12, 13, 14, 15, 16, 17, 18].

In 2017, the United States Food and Drug Administration (FDA) stated that biotin interference may cause erroneous test results, and this information was updated in 2019. The FDA has reported that biotin, found in many multivitamins, interacts with immunodiagnostic tests, including some tests for troponin, a clinically important biomarker that helps diagnose heart attacks. Despite these warnings, the FDA still receives adverse event reports of biotin interference and warns manufacturers about biotin interference [6, 19]. In clinical studies, biotinylated antibodies and antigens are frequently used in immunologic tests. It has also been shown that biotin taken at doses of 100–300 mg daily may adversely affect immunologic test results, such as cardiac and cancer marker data, and may cause erroneous results in many hormone parameters studied in automated immunoassay systems [20, 21, 22]. All immunoassays that use streptavidin‐biotin binding as part of the test reaction are susceptible to biotin interference from excess biotin in the blood. The mechanism of interference is related to signal loss in the immunoassay because high doses of biotin in the blood bind and block biotinylated reagents that are captured by the biotin binding sites on the streptavidin‐coated solid phase [7].

Despite the warning from the FDA, research on biotin interference is relatively new. Today, many laboratories are still unaware of interference problems or have not taken precautions concerning the extent to which biotin interference affects test results. There are very few studies showing biotin interference, especially in studies performed with research kits other than routine tests [23, 24]. None of these studies have been conducted in pediatric obesity. In our study, we aimed to examine the possible effects of different biotin concentrations on several research parameters in the case of pediatric obesity within the framework of the principles of creating ELISA kits and estimating possible biotin interference via these research tools.

## 2 Methodology

All laboratory studies were performed in the Biochemistry Research Laboratory of the university. Before the study, Ethics Committee approval documents numbered 2020.01.01.01 were obtained from the institution where the study was conducted. Families of the children, especially their parents, were informed. After a detailed pediatric physical examination and informing them about their inclusion in the study, signed consent forms from the consenting parents were attained and children were informed verbally. According to the World Health Organization (WHO) criteria with age‐ and sex‐specific body mass index percentile values above 95 and between 15 and 85, respectively, were included in the study. The study included healthy and obese children aged 5–18 years.

In this study, the concentrations of leptin, leptin receptor (LEPR), ghrelin, acylated ghrelin, deacyl ghrelin, ghrelin receptor (GHSR), kisspeptin (KISS1), kisspeptin receptor (KISS1R), preptin, peroxisome proliferator‐activated receptor gamma (PPARγ), Nod‐like receptor pyrin domain‐containing 3 (NLRP3), and interleukin‐18 (IL‐18) were determined using enzyme‐linked immunosorbent assay (ELISA) kits prepared with biotinylated reagents. Table 1 shows the detection ranges and CV values of the ELISA kits. All of the assays are Research Use Only (RUO) assays.

**TABLE 1:** Detection range and CV values of the ELISA kits.

ELISA Kits	Manufacturers	Catalog number	Intra assay CV (%)	Inter assay CV (%)	Detection range
Leptin sandwich	DRG	EIA‐2395	7.3%–7.1%–4.2%	6.9%–3.7%–9.1%	0.7–100 ng/mL
Ghrelin sandwich	Bioassay Technology Laboratory	E3091Hu	< 8%	< 10%	0.05–10 ng/mL
Acylated ghrelin sandwich	Bioassay Technology Laboratory	E3090Hu	< 8%	< 10%	0.05–15 ng/mL
Deacyl ghrelin sandwich	Bioassay Technology Laboratory	E4218Hu	< 8%	< 10%	0.5–200 ng/L
GHSR sandwich	Cloud‐Clone Corp.	SEC516Hu	< 10%	< 12%	0.156–10 ng/mL
Ghrelin competitive	Elabscience	E‐EL‐H1919	6%–4.44%–3.17%	6%–4.44%–3.51%	0.16–10 ng/mL
LEPR sandwich	Elabscience	E‐EL‐H0093	6.48%–5.43%–3.44%	6.25%–5.21%–3.49%	0.31–20 ng/mL
KISS1 sandwich	Elabscience	E‐EL‐H2129	5.1%–4.65%–4.9%	6.59%–4.94%–3.11%	125–8000 pg/mL
KISS1 competitive	Cloud‐Clone Corp.	CEC559Hu	< 10%	< 12%	12.35–1000 pg/mL
KISS1R sandwich	Cloud‐Clone Corp.	SEF540Hu	< 10%	< 12%	0.312–20 ng/ml
NLRP3 sandwich	Cloud‐Clone Corp.	SEK115Hu	< 10%	< 12%	0.312–20 ng/ml
Preptin sandwich	Elabscience	E‐EL‐H0913	5.62%–4.63%–4.65%	6.99%–5.24%–3.03%	62.5–4000 pg/mL
PPARγ sandwich	Elabscience	E‐EL‐H1361	6.38%–6.25%–3.48%	6.52%–6.09%–5.41%	0.16–10 ng/mL
IL‐18 sandwich	Elabscience	E‐EL‐H0253	6.04%–4.67%–4.81%	6.88%–4.37%–3.59%	15.63–1000 pg/mL
Biotin andwich	Bioassay Technology Laboratory	E3932Hu	< 8%	< 10%	1–300 ng/mL
Biotin Competitive	Cusabio	CSB‐E16209	< 8%	< 10%	12.5–200 pg/mL

Abbreviations: GHSR, ghrelin receptor; IL‐18, interleukin‐18; KISS1, kisspeptin; KISS1R, kisspeptin receptor; LEPR, leptin receptor; NLRP3, nod‐like receptor pyrin domain‐containing 3; PPARγ, peroxisome proliferator activated receptor gamma.

Serum pools were prepared from the blood samples of healthy controls and obese children. The study included 5 individuals in the control group (45 samples) and 5 individuals in the obese group (45 samples). A 1 mg/mL stock of biotin (Sigma‐14,400) solution with 0.1 mol/L NaOH was obtained. Using this stock solution of biotin, working solutions containing biotin at concentrations of 0 (control), 10, 25, 50, 100, 250, 500, 1000, and 1200 ng/mL were prepared by serial dilution with distilled water and were subsequently added to the serum samples. The biotin concentrations in the serum were determined by both the sandwich biotin ELISA kit and the competitive biotin ELISA kit. In biotin sandwich and competitive parameters, subgroups were named as obese 1 (O1), obese 2 (O2), obese 3 (O3), obese 4 (O4), obese 5 (O5), healthy control 1 (C1), healthy control 2 (C2), healthy control 3 (C3), healthy control 4 (C4), healthy control 5 (C5).

In the second phase of the study, working solutions containing biotin at concentrations of 10, 50, 500, and 1000 ng/mL were prepared from a 1 mg/mL stock biotin solution. The study was conducted from the serum pool prepared with samples taken from healthy children and obese children. A total of 28 samples, 3 groups (12 samples) from healthy individuals and 4 groups (16 samples) from obese individuals, were used in the study. The various working solutions were added to the pooled serum samples. Then, leptin, LEPR, ghrelin, acylated ghrelin, deacylated ghrelin, GHSR, KISS1, KISS1R, preptin, PPARγ, NLRP3, and IL‐18 concentrations were measured in each sample via the sandwich method. The ghrelin and KISS1 parameters were determined based upon both sandwich and competitive principles. The difference in Biotin % bias between the groups formed by the addition of biotin at concentrations of 10, 50, 500, and 1000 ng/mL was calculated with the following formula: measured value (concentration of the parameter with 50, 500 and 1000 ng/mL biotin added)—value not affected by biotin interference (concentration of the parameter with 10 ng/mL biotin added)/value not affected by biotin interference (concentration of the parameter with 10 ng/mL biotin added) × 100.

## 3 Statistics

All the statistical analyses were performed with SPSS (Statistical Package for the Social Sciences) Version 26. Parametric or nonparametric statistical tests were applied according to the distribution characteristics of the data. The Shapiro–Wilk test was used to determine whether the dataset followed a normal distribution. In addition to the independent samples t test, the Mann–Whitney *U* test and Kruskal–Wallis test were used to evaluate the differences between the groups, and Pearson and Spearman correlation analyses were performed to examine whether the values related to the parameters changed together. In addition, Bonferroni correction was used, and the Mann–Whitney *U* test was applied in the evaluation of subgroups. *p* < 0.05 were considered to indicate statistical significance.

## 4 Results

**FIGURE 1:**
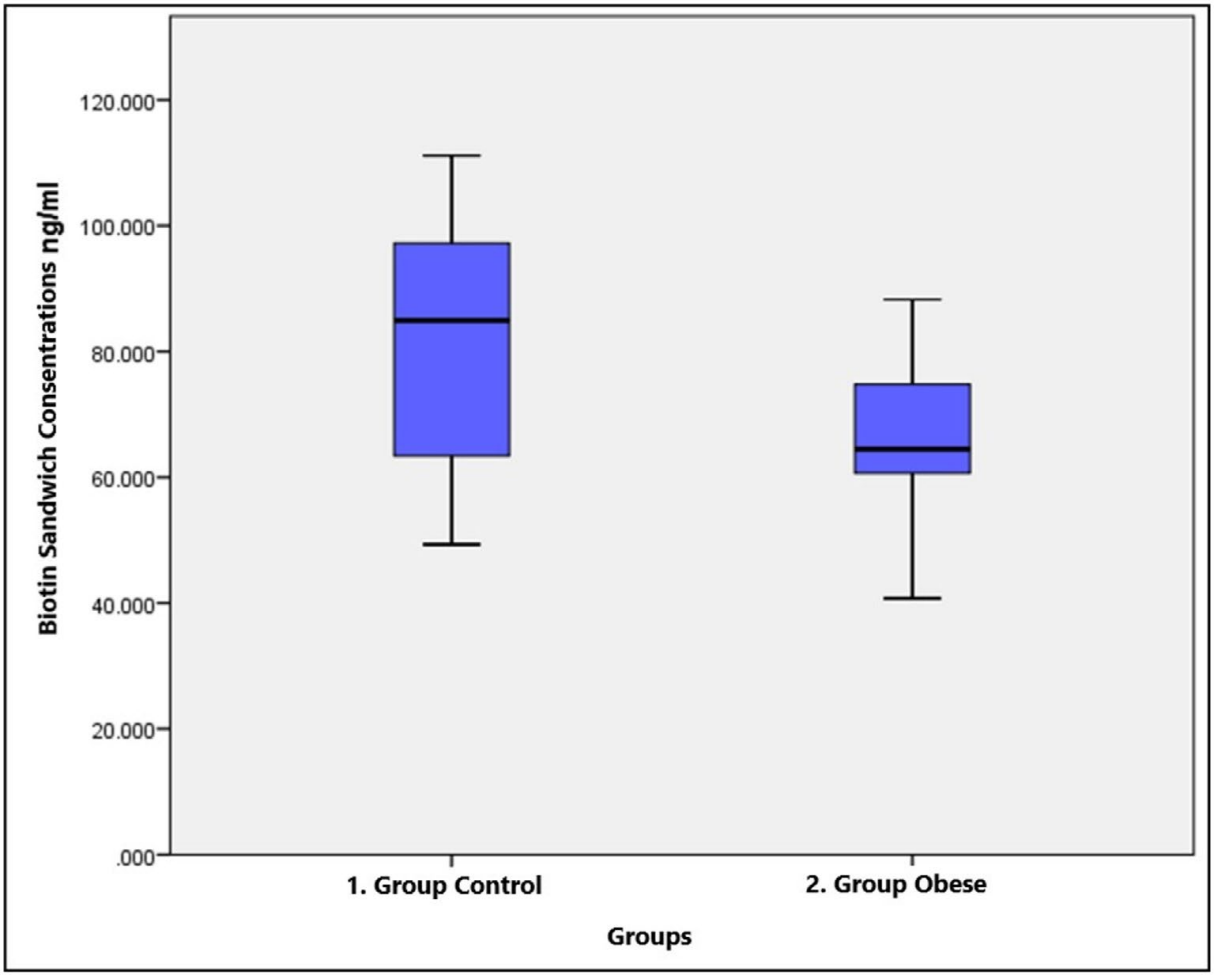
Concentration graph of biotin sandwich parameter in healthy control and obese children.

**FIGURE 2:**
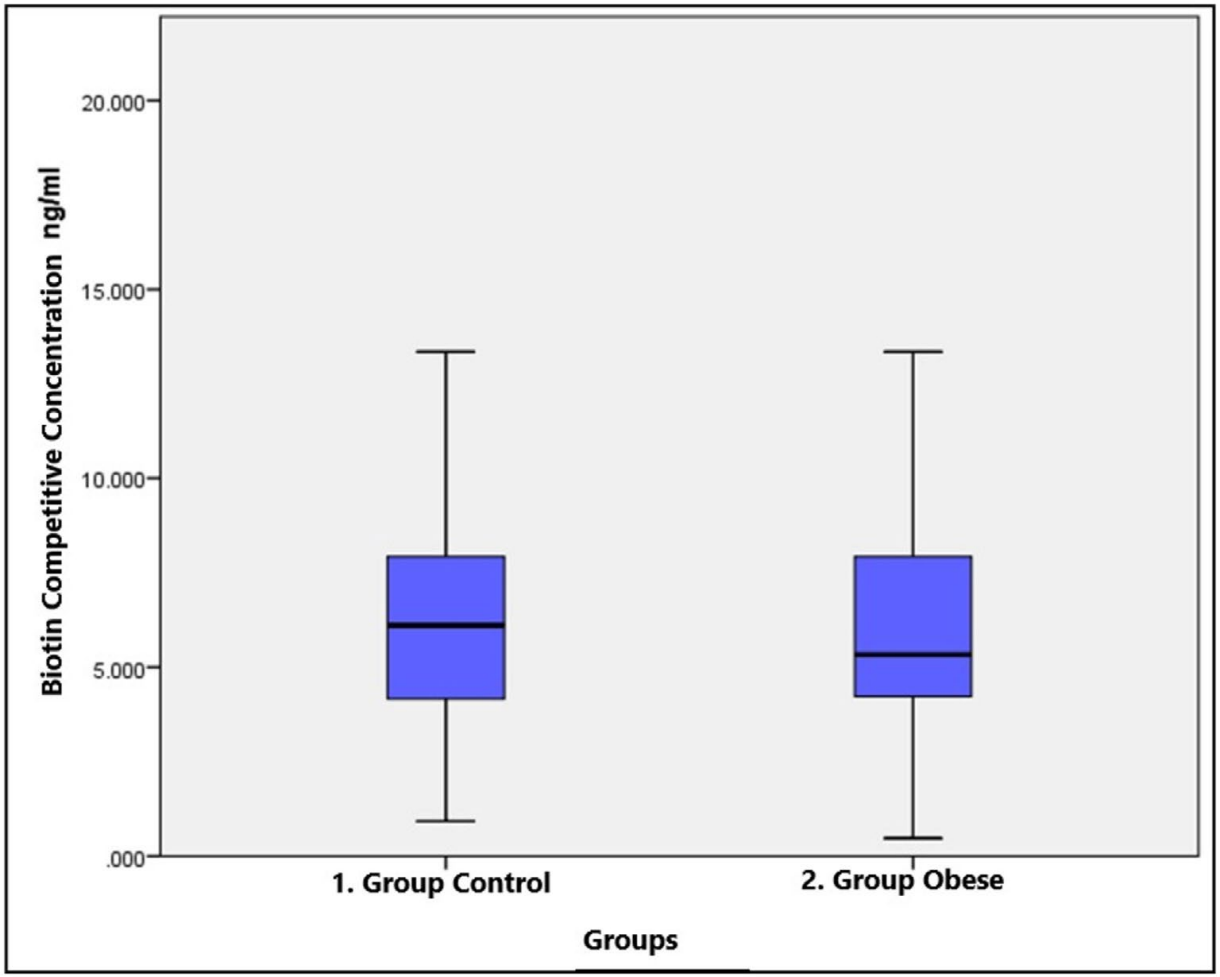
Concentration graph of biotin competitive parameter in healthy control and obese children.

**FIGURE 3:**
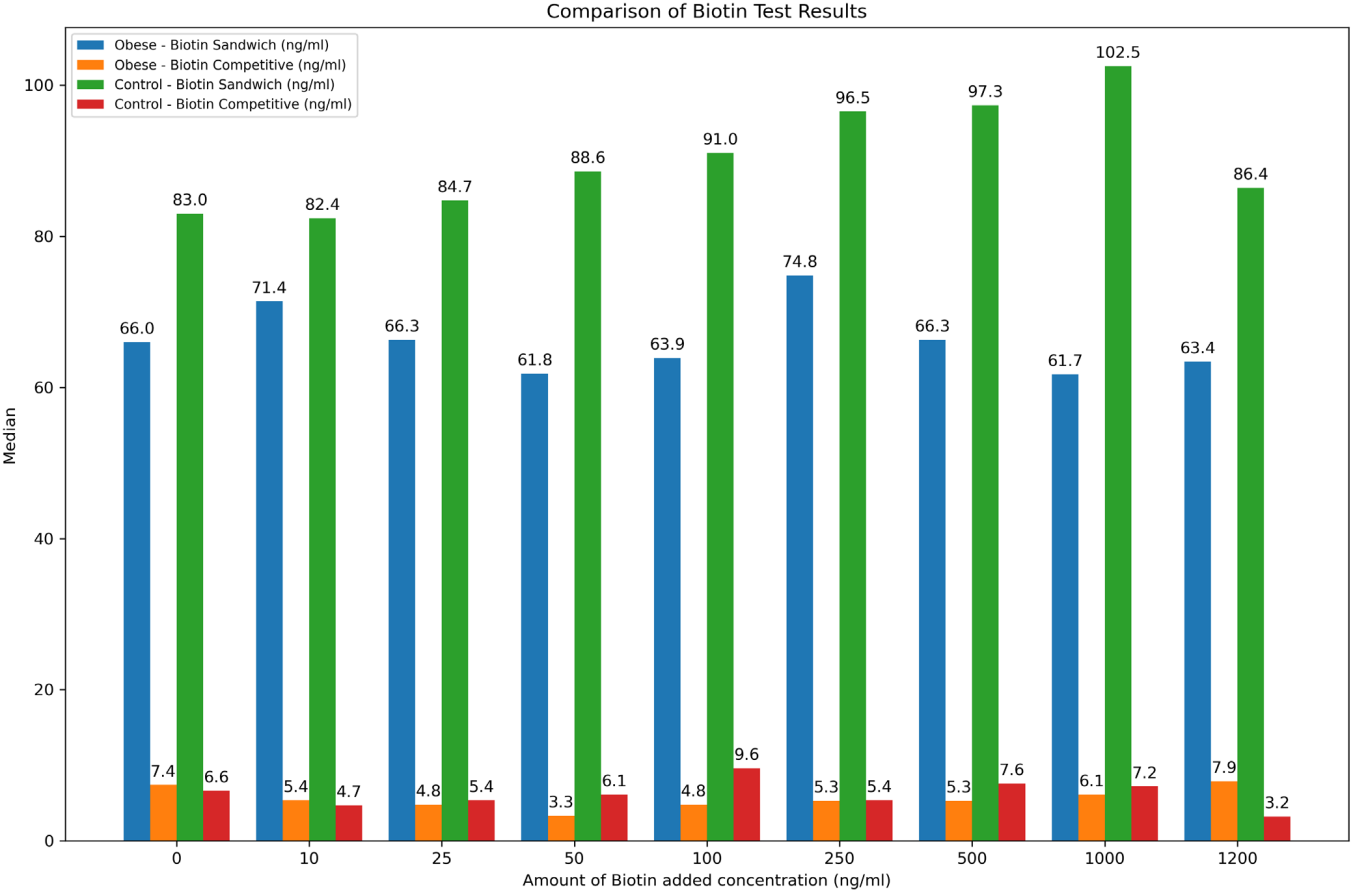
Median values of biotin sandwich and biotin competitive tests for obese and control groups.

The biotin concentrations of pooled serum samples containing 0 (control), 10, 25, 50, 100, 250, 500, 1000, or 1200 ng/mL biotin working solution were compared with those of two different ELISA methods, sandwich and competitive. In the biotin study of the healthy control and obese groups, the number of samples and the median, minimum–maximum and *p* values of the biotin parameter working with the sandwich and competitive methods are given in Table 2. The biotin sandwich parameter was significantly lower in the obese group (***p ≤ 0.001), while the difference between the groups was not significant for the biotin competitive parameter (*p* = 0.675). The biotin concentrations of healthy controls and obese children are shown in Figures 1 and 2. A significant positive correlation (*r* = 0.572; ***p < 0.001) was found between the values of the biotin sandwich and biotin competitive parameters in the control group, while no correlation was found in the obese group (*r* = −0.108; *p* > 0.05). Figure 3 shows the number of samples and median values of the control and obese groups according to the amount of added biotin. Moreover, there was no significant difference between the obese and control groups according to the amount of added biotin and the biotin sandwich parameters. Biotin concentrations measured by the sandwich ELISA method in children were found to be much greater than those measured by the competitive method. The number of samples, medians, and *p* values for the biotin parameter subgroups are shown in Table 3 and statistically significant differences among the groups were found for both the biotin sandwich (***p < 0.001) and biotin competitive parameters (**p < 0.01). In addition, Mann–Whitney *U* test with Bonferroni correction was performed as a *post hoc* test to reveal the difference between the subgroups of the biotin parameter. With Bonferroni correction, *p*:0.05/5 = 0.01 and all pairwise comparisons with *p* < 0.01 were considered significant. A significant difference (**p < 0.01) was found between the C1–O1, C2–O2, C3–O3, and C5–O5 groups in terms of the biotin sandwich parameter. While there was a significant difference (**p < 0.01) only between the C2–O2 groups in terms of the biotin competitive parameter, no significant difference was found in any other group.

**TABLE 2:** Samples, medians, minimum‐maximum values, and *p* values for the biotin parameter.

Parameters	Groups	Samples	Min.	Max.	Median	IQR	*p*
Biotin sandwich (ng/mL)	Control	45	49.3	111.1	84.9	30.90	
	Obese	45	40.7	88.2	64.4	23.75	0.001***
Biotin competitive (ng/mL)	Control	45	0.919	13.347	6.107	6.21	
	Obese	45	0.472	15.513	5.333	7.52	0.675

*Note:* The unit of the biotin competitive kit has been converted to ng/mL. *P≤ 0.05, **P≤ 0.01, ***P≤ 0.001.

Abbreviations: max, maximum; min, minimum.

**TABLE 3:** Samples, medians and *p* values for subgroups of the biotin parameter.

Parameters	Groups	Samples	Median	*p*
Biotin sandwich (ng/mL)	Obese 1 (O1)	9	79.9	
	Obese 2 (O2)	9	66.3	
	Obese 3 (O3)	9	62.2	
	Obese 4 (O4)	9	60.6	
	Obese 5 (O5)	9	60.6	
	Healthy control 1 (C1)	9	94.4	
	Healthy control 2 (C2)	9	97.2	
	Healthy control 3 (C3)	9	97	
	Healthy control 4 (C4)	9	68.2	
	Healthy control 5 (C5)	9	56.5	
	Total	90	70.2	0.000***
Biotin competitive (ng/mL)	Obese 1 (O1)	9	4.6	
	Obese 2 (O2)	9	4.6	
	Obese 3 (O3)	9	6.7	
	Obese 4 (O4)	9	8.8	
	Obese 5 (O5)	9	5.3	
	Healthy control 1 (C1)	9	7.1	
	Healthy control 2 (C2)	9	6.6	
	Healthy control 3 (C3)	9	8.2	
	Healthy control 4 (C4)	9	4.1	
	Healthy control 5 (C5)	9	4.4	
	Total	90	5.4	0.008**

**FIGURE 4:**
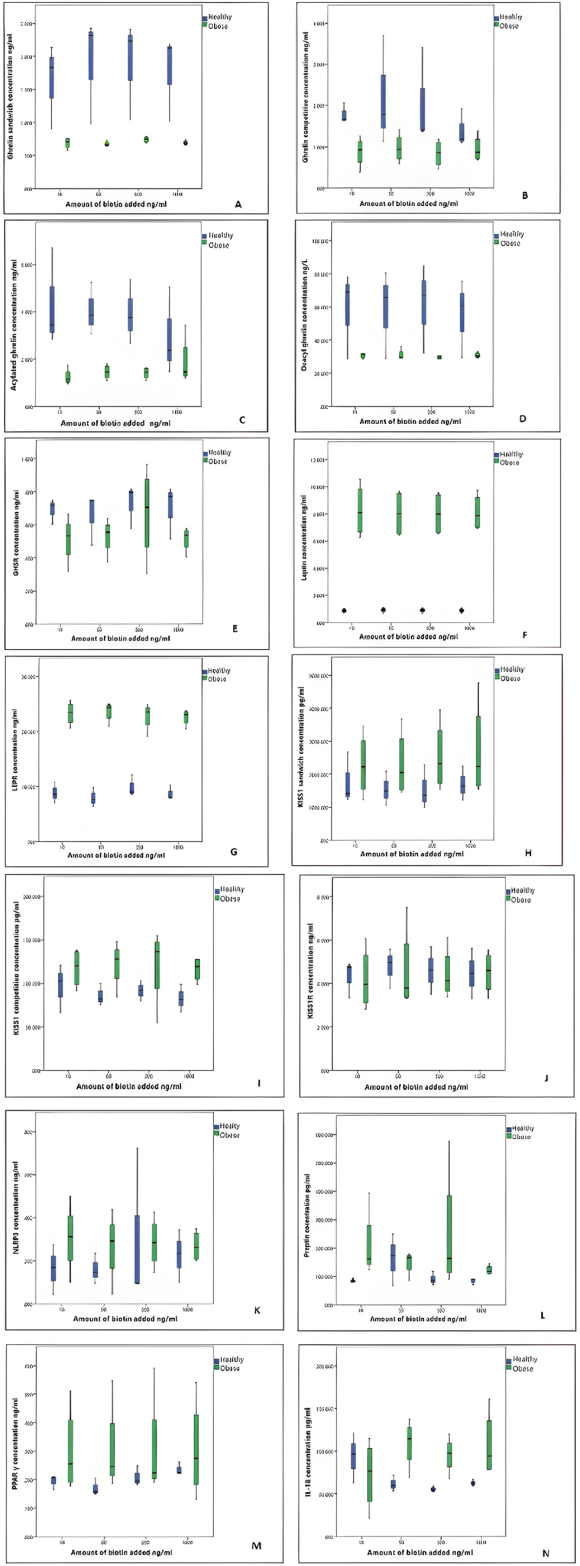
The changes in the concentrations of the parameters as a result of adding 10, 50, 500, and 1000 ng/mL biotin to all ELISA parameters in healthy and obese groups are shown in the graph. (A) The graph was shown ghrelin sandwich parameter biotin interference. (B) The graph was shown ghrelin competitive parameter biotin interference. (C) The graph was shown acylated ghrelin parameter biotin interference. (D) The graph was shown deacyl ghrelin parameter biotin interference. (E) The graph was shown GHSR parameter biotin interference. (F) The graph was shown for leptin parameter biotin interference. (G) The graph was shown LEPR parameter biotin interference. (H) The graph was shown KISS1 sandwich parameter biotin interference. (I) The graph was shown KISS1 competitive parameter biotin interference. (J) The graph was shown KISSR parameter biotin interference. (K) The graph was shown NLRP3 parameter biotin interference. (L) The graph was shown preptin parameter biotin interference. (M) The graph was shown PPARγ parameter biotin interference. (N) The graph was shown IL‐18 parameter biotin interference.

**FIGURE 5:**
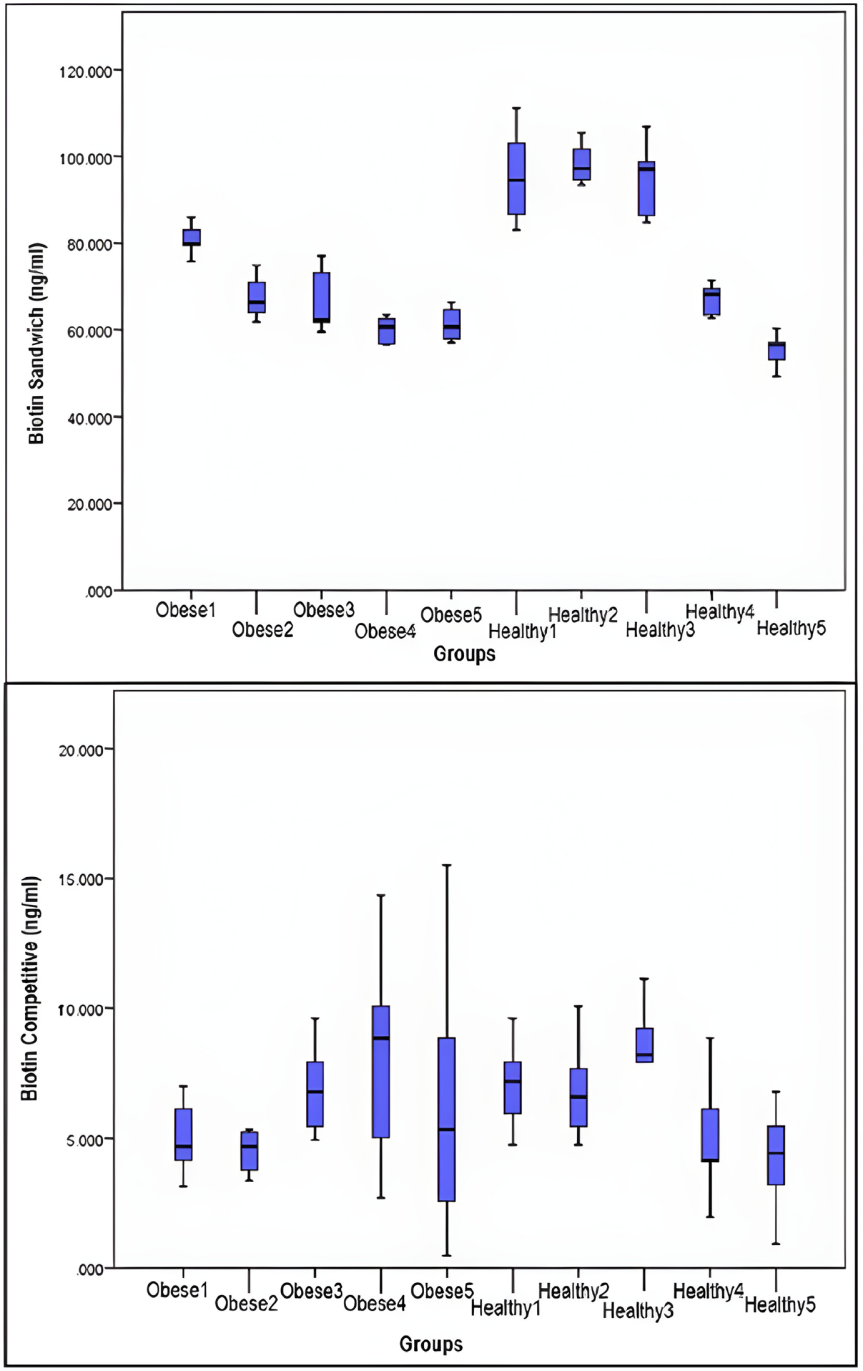
Biotin concentration in obese and healthy subgroups graph.

We conducted analyses to determine the interference effect of biotin at concentrations of 10, 50, 500, and 1000 ng/mL for NLRP3, leptin, ghrelin, acylated ghrelin, deacylated ghrelin, KISS1, preptin, PPARγ, LEPR, GHSR, KISS1R, and IL‐18. The number of samples, mean, SD, and median values of ELISA parameters according to the amount of biotin added at 10, 50, 500, and 1000 ng/mL between the healthy and obese groups are given in Tables 4 and 5. The results were stratified based on the distribution characteristics of the data. Table 4 presents the mean ± standard deviation (SD) values for parameters that followed a normal distribution, while Table 5 includes the median values and corresponding non‐parametric analyses for parameters that did not follow a normal distribution. This distinction was made to ensure appropriate statistical interpretation based on the underlying data characteristics. The % bias values of all parameters at 10, 50, 500, and 1000 ng/mL added biotin concentrations are shown in Table 6. See Figure 4A–N for changes in the concentrations of parameters due to the addition of 10, 50, 500, and 1000 ng/mL biotin to all ELISA parameters in the healthy and obese groups. The biotin concentration in obese and healthy subgroups is shown in Figure 5.

**TABLE 4:** Mean ± SD values of ELISA parameters for normally distributed data, according to biotin concentrations of 10, 50, 500, and 1000 ng/mL.

Groups	Amount of biotin added (ng/mL)		GHSR (ng/mL)	KISS1 competitive (pg/mL)	KISS1R (ng/mL)	Leptin (ng/mL)	NLRP3 (ng/mL)
Healthy	10	Mean	0.69	96.34	4.32	0.87	0.16
		Samples	3	3	3	3	3
		SD	0.07	27.7	0.8	0.16	0.11
	50	Mean	0.65	85.82	4.77	0.92	0.16
		Samples	3	3	3	3	3
		SD	0.15	12.66	0.90	0.21	0.07
	500	Mean	0.72	91.44	4.60	0.9	0.30
		Samples	3	3	3	3	3
		SD	0.13	11.78	1.09	0.20	0.36
	1000	Mean	0.7	82.45	4.46	0.88	0.22
		Samples	3	3	3	3	3
		SD	0.16	15.70	1.15	0.19	0.12
	Total	Mean	0.69	89.01	4.54	0.89	0.21
		Samples	12	12	12	12	12
		SD	0.11	16.42	0.87	0.16	0.18
Obese	10	Mean	0.51	117.25	4.20	8.25	0.30
		Samples	4	4	4	4	4
		SD	0.14	22.33	1.43	1.94	0.16
	50	Mean	0.53	121.88	4.59	8.02	0.26
		Samples	4	4	4	4	4
		SD	0.11	26.53	1.97	1.73	0.16
	500	Mean	0.67	120.51	4.44	8.01	0.28
		Samples	4	4	4	4	4
		SD	0.27	44.63	1.18	1.60	0.11
	1000	Mean	0.51	116.09	4.51	8.10	0.27
		Samples	4	4	4	4	4
		SD	0.07	13.94	0.98	1.33	0.07
	Total	Mean	0.55	118.93	4.44	8.09	0.28
		Samples	16	16	16	16	16
		SD	0.16	26.14	1.3	1.49	0.12

Abbreviations: GHSR, ghrelin receptor; KISS1, kisspeptin; KISS1R, kisspeptin receptor; NLRP3, nod‐like receptor pyrin domain‐containing 3.

**TABLE 5:** Median values and sample sizes of ELISA parameters for non‐normally distributed data, according to biotin concentrations of 10, 50, 500, and 1000 ng/mL.

Groups	Amount of biotin added (ng/mL)		Açylated ghrelin (ng/mL)	Deaçyl ghrelin (ng/L)	Ghrelin (ng/mL)	Ghrelin competitive (ng/mL)	IL‐18 (pg/mL)	KISS1 sandwich (pg/mL)	LEPR (ng/mL)	PPARγ (ng/mL)	Preptin (pg/mL)
Healthy	10	Samples	3	3	3	3	3	3	3	3	3
		Median	3.44	69.08	1.83	1.65	96.65	1417.28	8.64	0.20	80.83
	50	Samples	3	3	3	3	3	3	3	3	3
		Median	3.84	65.77	2.31	1.78	59.63	1490.34	7.64	0.16	172.63
	500	Samples	3	3	3	3	3	3	3	3	3
		Median	3.76	66.75	2.22	1.42	54.70	1362.13	9.06	0.19	85.17
	1000	Samples	3	3	3	3	3	3	3	3	3
		Median	2.37	60.91	2.12	1.18	62.48	1640.25	8.06	0.22	89.64
	Total	Samples	12	12	12	12	12	12	12	12	12
		Median	3.60	66.26	2.12	1.64	61.05	1453.81	8.53	0.20	87.40
Obese	10	Samples	4	4	4	4	4	4	4	4	4
		Median	1.15	31.17	0.70	0.92	76.77	2228.29	23.51	0.25	162.13
	50	Samples	4	4	4	4	4	4	4	4	4
		Median	1.45	29.73	0.66	0.93	114.55	2045.84	24.29	0.24	165.09
	500	Samples	4	4	4	4	4	4	4	4	4
		Median	1.44	29.77	0.75	0.85	97.67	2322.44	23.55	0.22	163.58
	1000	Samples	4	4	4	4	4	4	4	4	4
		Median	1.46	30.56	0.68	0.86	94.48	2233.3	23.04	0.27	116.92
	Total	Samples	16	16	16	16	16	16	16	16	16
		Median	1.31	30.18	0.68	0.92	97.67	2194.26	23.55	0.24	153.05

Abbreviations: IL‐18, interleukin‐18; KISS1, kisspeptin; LEPR, leptin receptor; PPARγ, peroxisome proliferator‐activated receptor gamma.

**TABLE 6:** The % bias values of all parameters at 10, 50, 500 and 1000 ng/mL added biotin.

Groups	Analyte	Analyte concentration (10 ng/mL added biotin)	% Bias with added biotin (ng/mL)		
			50	500	1000
Healty	Acylated ghrelin (ng/mL)	3.44	11.77	9.36	−30.95
	Deacyl ghrelin (ng/L)	69.08	−4.8	−3.37	−11.82
	Ghrelin (ng/mL)	1.83	26.47	21.71	15.91
	Ghrelin competitive (ng/mL)	1.66	7.59	−14.17	−28.5
	IL‐18 (pg/mL)	96.65	−38.3	−43.39	−35.35
	Leptin (ng/mL)	0.87	5.51	3.33	1.49
	LEPR (ng/mL)	8.64	−11.55	4.89	−6.68
	NLRP3 (ng/mL)	0.16	−2.45	85.88	38.65
	PPARγ (ng/mL)	0.20	−23.18	−5.31	8.21
	Preptin (pg/mL)	80.83	113.58	5.36	10.89
	GHSR (ng/mL)	0.69	−4.78	5.65	1.44
	KISS1 sandwich (pg/mL)	1417.28	5.15	−3.89	15.73
	KISS1 competitive (pg/mL)	96.35	−10.93	−5.09	−14.42
	KISS1R (ng/mL)	4.32	10.53	6.5	3.37
Obese	Acylated ghrelin (ng/mL)	1.15	26.45	25.76	27.32
	Deacyl ghrelin (ng/L)	31.17	−4.61	−4.48	−1.93
	Ghrelin (ng/mL)	0.70	−3.17	5.94	−2.69
	Ghrelin competitive (ng/mL)	0.92	1.18	−7.67	−6.37
	IL‐18 (pg/mL)	76.77	49.21	27.23	23.07
	Leptin (ng/mL)	8.25	−2.74	−2.94	−1.85
	LEPR (ng/mL)	23.51	3.31	0.17	−2.01
	NLRP3 (ng/mL)	0.30	−12.78	−6.88	−12.13
	PPARγ (ng/mL)	0.25	−39.2	−12.15	7.84
	KISS1 sandwich (pg/mL)	2228.29	−8.18	4.22	0.22
	KISS1 competitive (pg/mL)	117.25	3.94	2.78	−0.98
	KISS1R (ng/mL)	4.20	9.24	5.6	7.34
	Preptin (pg/mL)	162.11	1.83	0.9	−27.87
	GHSR (ng/mL)	0.511	3.91	31.31	0.78

Abbreviations: GHSR, ghrelin receptor; IL‐18, interleukin‐18; KISS1, kisspeptin; KISS1R, kisspeptin receptor; LEPR, leptin receptor; NLRP3, nod‐like receptor pyrin domain‐containing 3; PPARγ, peroxisome proliferator‐activated receptor gamma.

## 5 Discussion

Biotin, which is often found at high levels in multivitamins, including supplements used for hair, skin, and nail growth, can significantly interfere with certain laboratory tests and cause undetectable false high results [6]. In a study performed in children and adolescents, no relationship was found between dietary biotin intake or obesity and adiposity [25]. In addition, it has been shown that there was no change in blood biotin levels before or after bariatric surgery in obese adults [26]. In our study, no significant difference was found between the control and obese groups in terms of the biotin concentration studied via the competitive method.

Normal biotin levels in blood are lower in children than in adults. The normal range of serum biotin levels in children aged 0–4 years is 0.047–0.22 ng/mL, whereas it is 0.084–0.205 ng/mL in adults [27]. Normal serum biotin concentrations in children aged 6–12 years were found as the mean ± SD (0.14 ± 0.05 ng/mL) with a median of 0.1299 ng/mL [28]. In our study, there was a significant difference between the biotin concentrations measured by the biotin sandwich ELISA kit and the competitive ELISA kit. High biotin concentrations were observed with the sandwich ELISA kit, as shown in Table 2. The biotin values given in the above reports were evaluated. The range of normal biotin values in children's blood was compatible with the biotin values measured with the competitive kit. This finding showed that there may be severe interference in the biotin sandwich ELISA kit and that the biotin concentrations measured by the competitive method can provide more accurate results. The biotin sandwich kit used in our study does not have a pre‐dilution procedure. The fact that the measured parameter was directly biotin and that biotin was already used in the ELISA method caused a serious interference. However, the biotin‐competitive kit has pre‐dilution added to its procedure. This suggests that the biotin‐competitive kit was validated more accurately to reduce the interference effect and is therefore a better‐quality kit.

According to a report by the American Association for Clinical Chemistry (AACC), the results indicating biotin interference in laboratory tests included TSH and other thyroid function tests and endocrine tests such as PTH, ACTH, prolactin, testosterone, and cortisol. This report also described the biotin interference thresholds or biotin‐mediated interference levels of clinical tests for laboratory tests using immunologic methods by many manufacturers, such as Beckman Coulter Inc., Roche Diagnostics, Ortho Clinical Diagnostics, and Siemens [29].

In 2016, according to the evaluation of the 8 most popular immunoassay analyzers used in the United States, it was shown that exogenous biotin in the sample can cause more than ±10% biotin interference in test results from sandwich and competitive methods [30]. In another study, it was shown that high‐dose biotin caused false high results in cortisol, cyclosporine A, digitoxin, tacrolimus, FT3 and FT4 competitive immunological tests and false low results in C‐peptide, insulin, TSH, NT‐proBNP, high‐sensitivity troponin T, HIV, procalcitonin and b‐HCG sandwich immunological tests [31]. False negative results in troponin T tests are vital for patients, and newer cardiac troponin T test formulations are now being sought to have a much higher biotin interference threshold [32]. In a study of acute myocardial infarction patients, the probability of biotin interaction giving false negative results to the Elecsys Troponin T Gen 5 (TnT Gen 5; marketed outside the United States [US] as Elecsys Troponin T‐high sensitive; Roche Diagnostics International Ltd., Rotkreuz, Switzerland) test was found to be very low [33]. Dilution and biotin removal procedures could be used to reduce biotin interference in immunoassays [34].

Bonferroni correction and the Mann–Whitney *U* test were applied to evaluate the subgroups, as shown in Table 3. According to the biotin sandwich ELISA results, biotin levels were generally greater in the healthy group than in the obese group, while in the 5th group, biotin levels were greater in the obese group than in the healthy group. With respect to the biotin competitive parameter, in some subgroups, the value in the healthy group was greater than that in the obese group, whereas in the other groups, the value was greater than that in the healthy group. There was an apparent discrepancy. This finding suggested that biotin interference in the kits may lead to erroneous results. Kabiri et al. demonstrated that biotin interference in automated systems can lead to both false low and false high results in the sandwich method [35]. Although it is widely reported that subphysiologic doses of biotin typically cause false‐positive results in competitive assays and false‐negative results in sandwich assays [31, 36], our spike‐and‐recovery analyses demonstrated that both false low and false high results may occur in both assay formats when using manual ELISA kits. This finding suggests that biotin interference may not always follow the expected pattern, particularly in manually operated systems, and highlights the importance of assay‐specific validation when interpreting results in the presence of biotin.

Dasgupta et al. demonstrated that in the Cobas E‐411 (Roche Diagnostics) immunoassay IL‐6 test, maximum negative interference was observed with 100 ng/mL biotin, but no interference occurred with 1000 ng/mL biotin [37]. Knudsen et al. demonstrated that aldosterone and renin analyses performed in automated systems showed a very high rate of interference with biotin at doses of 100–500 ng/mL, while insulin‐like growth factor 1, growth hormone, and bone alkaline phosphatase analyses were less sensitive to biotin doses in this range [38]. Choi et al. showed that biotin interference is still present in second and third‐generation Elecsys (Roche Diagnostics) free thyroxine immunoassay tests updated by the company [39]. Biotin levels are not measured in all laboratories, which makes the problem of biotin interference difficult to solve. Dilution tests and neutralization analyses may help to solve this problem [40]. Numerous studies have demonstrated biotin interference in routine laboratory tests. This poses a serious risk for patients in the diagnosis and treatment processes.

In this study, we adhered to standard practices in interference testing by comparing results from spiked samples with those from unspiked baseline samples. For the biotin ELISA kits, we used a true 0 ng/mL biotin concentration as the baseline and assessed the effects of increasing biotin levels (10, 25, 50, 100, 250, 500, 1000, and 1200 ng/mL). In contrast, for the other ELISA‐based assays, we began spiking at 10 ng/mL rather than 0 ng/mL. This decision was influenced by practical limitations, including restricted sample volume and budget constraints. Although the threshold for biotin interference can vary depending on the type of immunoassay used, previous studies have reported that 10 ng/mL is a sufficient concentration to induce measurable interference in automated systems [29, 41]. Based on this evidence, we selected 10 ng/mL as the lowest spiking concentration for these assays. While this approach allowed us to focus on clinically relevant concentrations, we acknowledge that the absence of a true zero baseline in these cases is a limitation. Similar to our study, Katzman et al. evaluated biotin interference in the waste blood of adult patients. They measured biotin concentrations in blood using liquid chromatography tandem mass spectrometry and showed that patients were taking high levels of biotin supplements, which caused severe biotin interference in many immunoassays [41]. One of the limitations of our study is that we did not have the opportunity to use mass spectrometry due to budget constraints. However, conducting the study in children and demonstrating that biotin interference may also occur in research kits, especially in obesity parameters, will make a significant contribution to the literature.

For all the ELISA parameters, biotin % bias values between the groups formed by the addition of biotin at concentrations of 10, 50, 500, and 1000 ng/mL were calculated [4, 33, 35]. In our study, there was biotin interference in all ELISA parameters commonly used in investigations performed in the field of pediatric obesity, and this interference was found to be greater in the healthy group. In addition, the addition of 50, 500, or 1000 ng/mL biotin to the studied parameters created both negative and positive interferences. The % bias values for all parameters ranged between 1.4% and 113.6% for the healthy group and 0.17% and 49.2% for the obese group. For example, for all three biotin concentrations of the leptin kit, the % bias value in both the healthy and obese groups was < 5.6, the % bias value of the GHSR kit in the healthy group was < 5.7, the % bias value of the LEPR kit in the healthy group was < 11.6, and that in the obese group was < 3.4. The limitations of our study are that not all parameters can be compared with both sandwich and competitive methods, and the study cannot be conducted with all ELISA brands on the market, and the sample size is limited. This is because there are no ELISA kits on the market that use the competitive method for all the parameters we examined, and the budget is limited. Because of the relationship between biotin and microbiome diversity in severe obesity, giving biotin supplements to obese individuals may increase the risk of biotin interference in immunoassays. It is very important that our study is the first study showing biotin interference in pediatric obesity. It will contribute to raising awareness among scientists and healthcare professionals on this issue. Specifically, biotin interference is closely related to the quality of the ELISA kit, the parameters studied, and the concentration of biotin in the blood. If manufacturers review the quality control procedures, accuracy, recovery, and reproducibility analyses for interference in ELISA kits during the validation phase and make the necessary applications to prevent interference independently of cost calculations, this will lead to the production of higher quality kits. Thus, researchers can perform more accurate analyses and contribute to science.

## 6 Conclusion

In addition to ELISA kits used in routine practice, it has been shown that biotin interference can also occur in manual ELISA kits used in scientific research and can cause erroneous test results. Therefore, patients should be asked whether they use biotin supplements during scientific studies or routine analyses, and precautions should be taken to reduce the effect of biotin interference. ELISA kit manufacturers should take biotin interference into account. In order to reduce interference in kits, pre‐dilution, use of biotin‐binding nanoparticles, measurement of biotin concentration in samples with rapid tests, and ELISA kit manufacturers taking precautions by determining the cut‐off values of biotin concentrations in blood that create biotin interference may be useful.

In order to create a handbook to reveal the negative effects of biotin in laboratory studies, manual ELISA kits with many different brands and different parameters should be used, and the results obtained should be compared and supported with advanced techniques such as polymerase chain reaction (PCR) or mass spectrometry. Only in this way will it be possible to make more sensitive and accurate measurements in the analysis of many studies conducted to contribute to science.

## Author Contributions

All the authors have accepted responsibility for the entire content of this submitted manuscript and approved submission.

## Ethics Statement

Prior to the study, ethics approval was obtained from the institutional Ethics Committee (Approval No: 2020.01.01.01).

## Consent

The families of the participating children, particularly their parents, were informed about the study. Following a detailed pediatric physical examination and explanation of the study procedures, written informed consent was obtained from the parents, and verbal assent was obtained from the children.

## Conflicts of Interest

The authors declare no conflicts of interest.

## Data Availability

Due to ethical and privacy considerations, the data are not publicly available.
